# Theoretical Investigation of Forming Process of Aluminum Alloy Rail Vehicle Side Window

**DOI:** 10.3390/ma15238290

**Published:** 2022-11-22

**Authors:** Zhengwei Gu, Hongru Chen, Lingling Yi, Ziming Tang, Meng Wang, Ge Yu

**Affiliations:** 1 School of Materials Science and Engineering, Jilin University, Changchun 130022, China; 2 State Key Laboratory of Automobile Materials, Jilin University, Changchun 130022, China; 3 Faw-Volkswagen Automotive Co., Ltd., Changchun 130011, China

**Keywords:** superplastic forming, 5083Al, rail vehicle side window, RSM

## Abstract

With the vigorous development of rail transit trains around the world and the emergence of global environmental pollution and energy shortages, the world has an urgent need for manufacturing technology for lightweight aluminum alloy rail transit train components. This paper mainly studied the superplastic forming law of 5083Al for rail transit. Through the high-temperature tensile test and blowing forming experiments, the superplastic properties of 5083Al were determined. Based on this, the die design, finite element simulation, and forming experiment of the rail vehicle side window were carried out. In order to study the superplastic deformation behavior of industrial 5083Al under complex stress conditions, the influence of the depth, area ratio, and friction coefficient of the pre-forming die on the part thickness distribution is simulated. The side window is made of a high-strength 5083Al sheet in the form of bending at both ends to ensure the strength of the connection between the overall side window and the side wall skeleton. The variation law of the side wall forming height of 5083Al box-shaped parts was studied. The efficient manufacture of parts that meet quality standards was made possible by the optimization of the pressure profile. The microstructure changes of the material after superplastic forming were studied by Energy Dispersive Spectrometer (EDS) and Electron Backscattered Diffraction (EBSD).

## 1. Introduction

The high-speed development of rail transit gives rail transit faster speed and better social benefits. It has the advantages of energy saving, environmental protection, comfort, a high safety factor, and competition with other means of transportation. With the improvement of the transportation speed of rail vehicles, its lightness highlights its importance. The superplastic materials used in industrial production are mostly magnesium-based, titanium-based, and aluminum-based alloys. The obtained forming parts have the characteristics of high precision and small spring back and have significant advantages in forming parts with complex contours and large dimensions. An aluminum alloy is quite different from traditional steel, and the forming experience cannot be completely applied. Therefore, the aluminum alloy forming process is relatively complex, and the cost is high, which is one of the reasons that hinder the widespread use of aluminum alloys.

The superplastic forming process would definitely play an important role in the healthy and sustainable development of the rail transit industry. Megumi et al. believed that by applying severe plastic deformation to metal processing, it is possible to achieve smaller grain sizes on the submicron or even nanometer scale, which could provide an opportunity for new research on the superplastic flow [1]. In order to determine the suitability of the AZ41 magnesium alloy during superplastic forming and determine the optimal forming parameters, Taylor et al. studied the AZ41 alloy in the hot-rolled state. The alloy does not require further thermomechanical processing to change its microstructure [2]. Yang et al. established a physics-based internal state variable constitutive model (ISV) to describe the flow stress and microstructure evolution of Ti-6Al-4V alloy during superplastic forming [3]. Mosleh et al. proposed a comprehensive method for the superplastic forming of Ti-6Al-4V titanium alloys [4]. Li et al. adopted the superplastic forming/diffusion bonding (SPF/DB) process manufacturing and finite element to simulate and study the forming process and mechanical behavior of different topological structures [5]. However, the superplastic forming proposed by them is not very mature. Therefore, this paper introduced 5083Al and optimized its process.

The superplastic differential temperature tensile simulation of 5083Al was carried out to provide a basis for future industrial production. Majidi et al. studied the *m* value of superplastic grades of aluminum alloys (AA5083) [6]. Patel et al. believed that friction stir processing with the active cooling method can enhance the superplasticity of aluminum alloys [7]. Liao et al. studied the deformation behavior and mechanism of 7B04 aluminum alloy during superplastic deformation through high-temperature tensile tests, electron backscatter diffraction (EBSD), scanning electron microscopy (SEM), and transmission electron microscopy (TEM). The contribution of each deformation mechanism was calculated by a focused ion beam (FIB) [8]. Hosseini M E et al. believed that cavitation occurs in many materials during superplastic deformation. The strain rate sensitivity index of commercial 5083Al alloy material was determined by the superplastic bulging test [9]. The research of Qi et al. avoided the crack defect caused by anisotropy during the superplastic forming of 5083Al [10]. However, their proposed 5083Al alloy processing technology is not very good, and their research on the influence of superplastic forming quality on machining processes is not comprehensive enough.

In this paper, the forming properties of the 5083Al sheet and the flow law at different temperatures were studied, which provided a scientific basis for developing a superplastic forming technique for rail vehicle side windows. By adopting the method of rapid superplastic forming and numerical simulation technology, the influence of the depth, area ratio, and friction coefficient in the pre-forming die on the forming quality was investigated. After obtaining the best parameters of the pre-forming die, the forming of the local fillet was further optimized by varying the pressure profile. The effect of temperature and strain rate on the forming of aluminum alloy sheets was investigated. The forming limits of aluminum alloy sheets were analyzed by applying superplastic forming to box-shaped parts. The relationships obtained between temperatures, forming rates, and forming limits provided a technical reference for practical production. Energy spectrum scans showed little change in the phase types of the matrix and precipitated phases. The changes in the microstructure of the blank before and after blow forming were examined by EBSD techniques.

## 2. Aluminum Alloy Rail Vehicle Side Window Forming Design

### 2.1. Materials Model

The material used for the rail vehicle side window in this paper is industrial 5083Al alloy with a thickness of 4 mm, which has the characteristics of high strength, good corrosion resistance, good diffusion bonding performance, and low cost. The chemical composition is shown in Table 1.

**Figure 1:**
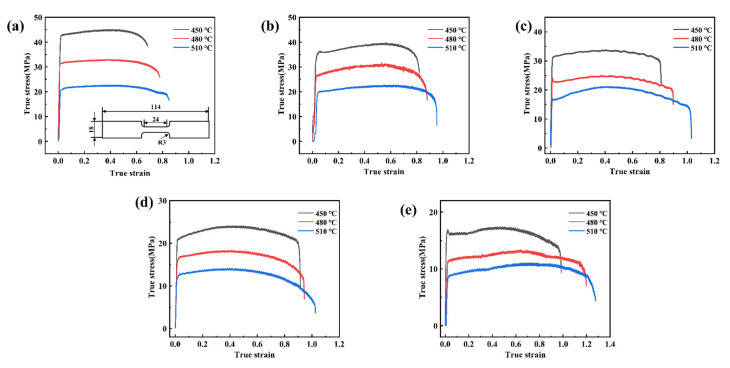
Flow stress curves at different strain rates and the shape and dimensions of the tensile specimen (**a**) $\dot{\varepsilon }=1\times 10^{-2}\;s^{-1}$, (**b**) $\dot{\varepsilon }=7.5\times 10^{-3}\;s^{-1}$, (**c**) $\dot{\varepsilon }=5\times 10^{-3}\;s^{-1}$, (**d**) $\dot{\varepsilon }=1\times 10^{-3}\;s^{-1}$, (**e**) $\dot{\varepsilon }=5\times 10^{-4}\;s^{-1}$.

High-temperature tensile tests were carried out. Tensile specimens are obtained by cutting in the direction of the rolling of the sheet, which is with a length of 800 mm and a width of 450 mm. The temperature was selected as 450 °C, 480 °C, and 510 °C respectively and the strain state were set as 1 × 10^−2^ s^−1^, 7.5 × 10^−3^ s^−1^, 5 × 10^−3^ s^−1^, 1 × 10^−3^ s^−1^, and 5 × 10^−4^ s^−1^. The flow stress curves at different strain rates and the shape and dimensions of the specimens for tensile tests are shown in Figure 1. From the graph, it can be obtained that the flow stress of the sheet with the deformation increases rapidly to the ultimate stress. As the deformation continues to increase, strain hardening interacts with strain softening, and the flow stress remains stable or decreases slightly. When the strain exceeds a critical value, the flow stress rapidly decreases, and the specimen eventually fractures. The maximum elongation is 196.1%, 209.4%, and 221.7% at deformation temperatures of 450 °C, 480 °C, and 510 °C, respectively. The optimum deformation conditions are a temperature of 510 °C and a strain rate of 5 × 10^−4^ s^−1^ when the elongation is 221.7%. 510 °C is already the limit value. If other factors are added, the length will not increase and may break.

### 2.2. Rapid Superplastic Forming of Rail Vehicle Side Window

Rapid superplastic forming (RSF) is a combination of hot stamping and direct superplastic forming (DSF) processes. RSF is not only characterized by rapid forming but can also precision form complex shaped parts. Applying this process, the optimum combination of parameters was found by adjusting the depth, area ratio, and friction coefficient of the pre-forming die to effectively reduce the thinning rate [11]. Pre-forming the blank using a hot stamping die with a specific shape allows more of the blank to enter the cavity, increasing the area of the blank involved in gas forming.

The relationship curve between the load of the side window and the displacement of the fixture can be obtained during the uniaxial tensile test. Through the connection between the chuck position and the gauge length, the actual elongation of the gauge length of the specimen is converted, and then the true stress σ and the true strain ε of the side window are calculated.

(1) σ=s(1+e)

(2) ε=ln(1+e)

where s is the engineering stress and *e* is the engineering strain.

The relationship between flow stress and strain rate of aluminum alloy rail vehicle side window can be expressed by a general constitutive function:

(3) $$\dot{\varepsilon }=A[\sinh(\alpha \sigma )]\exp(-\frac{Q}{RT})$$

where *A* is the material constant, α is the correction factor, *Q* is the heat-activated energy, *R* is the gas constant, and *T* is temperature.

**Figure 2:**
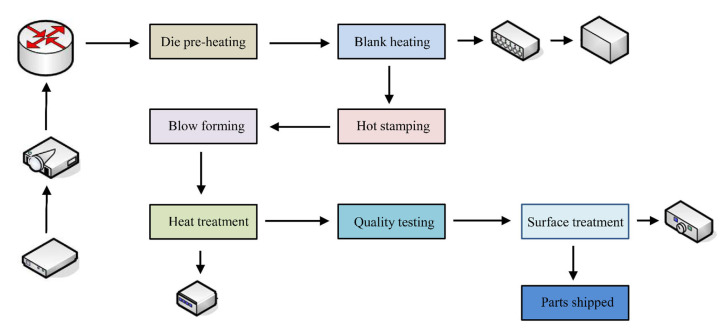
The process of the whole production.

Due to the consideration of the actual production situation, the superplastic forming process of aluminum alloy parts is divided into the following stages. The first is the sheet and die heating stage. It reflects the thermal expansion process of the sheet and the die from room temperature to the optimum forming temperature. The second is the sheet metal forming stage. The sheet is formed under the combined action of blankholder force and air pressure. The size of the pressure and the parameters of the die have a great influence on the microstructure changes during the deformation process and the final wall thickness distribution of the part. The third is the cooling stage of the part after forming. In practice, the parts are taken out and placed in air to cool after forming. Therefore, at this stage, the cooling process of the part is determined so that the sheet metal would shrink, and the final wall thickness distribution after forming can be obtained. The rapid superplastic forming process is divided into four steps: Alloy heating, pre-forming, blow forming, and heat treatment. The process of the whole production is shown in Figure 2 [12,13].

### 2.3. Stamping Effect of Aluminum Alloy Rail Vehicle Side Window

The magnitude of the flow stress exhibited by the 5083Al alloy sheet during the warm and thermal deformation process is related to the dislocation density generated in the deformation zone inside the material matrix. At the beginning of the deformation of the material, due to the entanglement of the interaction between dislocations generated inside the material, the phenomenon of work hardening occurs in the material [14]. When the deformation rate of the material increases or the degree of deformation increases, the dislocation density inside the material is larger and more concentrated, which finally leads to an increase in the flow stress of the material. The main reason for this phenomenon is that when the deformation temperature of the material is below 200 °C, and the deformation rate of the material is greater than 0.001 s^−1^, the internal structure of 5083Al alloy undergoes strong dynamic recovery during the deformation [15]. The relationship between the stress and strain of the aluminum alloy rail vehicle side window material satisfies the Backofen function:

(4) $$\sigma =k{\dot{\varepsilon }}^{m}$$

where σ is the flow stress, $\dot{\varepsilon }$ is the strain rate, *k* is the material constant, and *m* is the strain rate sensitivity index. When *m* is greater than or equal to 0.3, the material can be called a superplastic material, so the *m* value is an important index to judge whether the material is a superplastic material. At a specific strain, the *m* value is usually defined as:

(5) $$m=\frac{\partial \ln\sigma }{\partial \ln\dot{\varepsilon }}$$

It can be transformed into:

(6) $$\ln\sigma =\ln k+m\ln\varepsilon_{p}$$

The maximum strain rate value ${\bar{\varepsilon }}_{\max}$ of the aluminum alloy rail vehicle side window is:

(7) $${\bar{\varepsilon }}_{\max}=\max\{\varepsilon_{t}\}$$

where $\varepsilon_{t}$ is the value of the corresponding part strain. In the blow forming, the pressure provided by the forming must ensure the final die attaching of the sheet; that is, the die attaching with the smallest fillets must be ensured. In the experiment, the maximum forming pressure required when the side window is formed is approximately calculated according to the formula of the minimum fillet forming pressure:

(8) $$p=\frac{\delta_{s}t}{r}$$

where s is the corresponding unit area. The calculation method of the reduction of section *δ* in this paper is as follows: Firstly, the broken sample is spliced according to the fracture surface; the width of its narrowest part and the thickness at the fracture edge are measured with a dial caliper in turn, and then the minimum cross-sectional area *A*_1_ of the broken sample is calculated. Secondly, according to the original cross-sectional area *A*_0_ of this place, the section shrinkage rate *δ* of the sample is calculated. The calculation formula is:

(9) $$\delta =\frac{A_{0}-A_{1}}{A_{0}}$$

The thickness anisotropy coefficient *r* of the aluminum alloy rail vehicle side window sheet is taken as the average value of the data obtained from three samples in different directions and is calculated by Equation (10):

(10) $$r=\frac{r_{0}+2r_{45}+r_{90}}{4}$$

It is generally believed that the maximum thinning rate should be less than 40% after the side window is formed. In another way, the evaluation function is the uniformity of the thickness distribution of the blank after forming, and its expression is as follows [16]:

(11) $$J_{i}=\sum \begin{array}{l} n\\ i=0 \end{array}\frac{t_{i}}{t_{0}}$$

where *n* is the total number of nodes.

The dynamic recovery is due to the occurrence of cross-slip cut order, unconserved climbing motion, and disentanglement of the dislocation network in the internal microstructure of dislocation when the material undergoes warm-plastic deformation. The energy required for dislocation to generate dislocation slip is increased, so the deformation activation energy of 5083Al alloy during deformation is much higher than that of pure aluminum. When the 5083Al alloy is deformed, the internally generated screw dislocations will cross-slip. In this process, the screw dislocations would cross each other and finally form a cut order, and many vacancies or void atoms would be generated. Edge dislocations climb when the density of vacancies in the matrix increases. The higher the vacancy density, the easier it is to climb. When the degree of deformation of the material continues to increase, more and more dislocations are formed inside, which leads to an increasing dislocation density. When it increases to a certain extent, it will exceed the limit range that the material can bear, and it would appear as necking and cracking of the material on the macroscopic level [17]. The 5083Al alloy is more prone to dynamic recovery in the temperature range from room temperature to 200 °C, which reduces the dislocation density inside the aluminum alloy and increases the limit deformation degree. Moreover, the lower deformation rate can provide more opportunities for the internal dislocation slip of the aluminum alloy to reduce the dislocation density and the flow stress, thereby increasing the deformation limit of the aluminum alloy.

The hot stamping process is used for pre-forming; the higher the temperature, the lower the forming rate, which can increase the recrystallization driving force of the aluminum alloy material. When tension is applied to the blank, in the process of pulling the material, the weaker area in the blank is the interface between the die wall and the punch. However, if in the process of applying a tensile force to the material, the tensile force exceeds the stress of the thinnest material, the material would be scrapped due to the excessive tensile force. Typically, the problem to be prevented in deep drawing of materials is cracking and wrinkling [18]. Whether cracking and wrinkling can occur in the deep drawing is related to the thickness value of sheet metal and the degree of deformation. If the blankholder force is too large, the friction force between the blank and the die will increase, which would lead to pulling cracking. If the blankholder value is too low, the sheet will wrinkle due to the low stability of the sheet. It is considered that the blankholder value should be adjusted in the early stage of stamping, and the value range should be adjusted with the technical improvements. The geometry can also play a role in regulating the flow of the material, and the reasonable design of the die fillet radius can make the stress-strain process connection tend to be smooth. For ordinary body panel stamping, in order to avoid wrinkling, the yield limit of the stamping material should be reduced, the hardening index should be increased, and the elongation should be increased.

## 3. Exploration Results of Side Window Superplastic Forming

### 3.1. Simulation of Blow Forming of 5083Al Alloy

(1) Finite element model design

**Figure 3:**
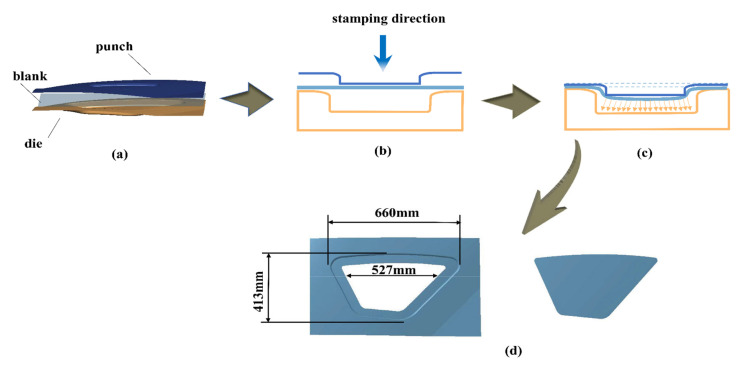
The forming process and geometry of the rail vehicle side window (**a**) Finite element model of the forming process (**b**) Hot stamping process (**c**) Blow forming process (**d**) Part geometry.

The forming process and geometry of the rail vehicle side window is shown in Figure 3. The depth of the side window is 38.2 mm, and the sharp, rounded bottom on the right side is the most difficult part to form and the part that is most prone to cracking during the forming process. A finite element model was established according to the geometry of the side window, and the superplastic forming analysis process was simulated by the finite element analysis software pam-stamp. The die face and punch face were defined as non-deformable rigid bodies, and the industrial 5083Al sheet should be defined as deformable bodies. The sheet is discretized using Belytschko-Tsay (BT) shell cells with the same mesh size. The m and k values for the material are 0.331 and 156.3, respectively. The friction coefficient of the whole process was set to 0.15 (friction coefficients used in numerical simulations of superplastic forming generally range from 0.1 to 0.2), and it was placed horizontally on the die. In the first step, the punch is driven down while the die is fixed, completing the pre-forming of the sheet. The second step is to blow forming on the basis of the blank fit to die. During the forming process, all six degrees of freedom of the nodes around the sheet are fully locked, and the part is shaped by the injection of gas. The unit volume of the sheet was calculated from the volume between the surface element and the projection on a given plane and adopted the nonlinear viscoelasticity law as its flow law. Through the action of the pump or the movement of the tool, with the increase of pressure, the deformation of the part is completed, and the formed part is finally obtained.

(2) Determination of part forming process

**Figure 4:**
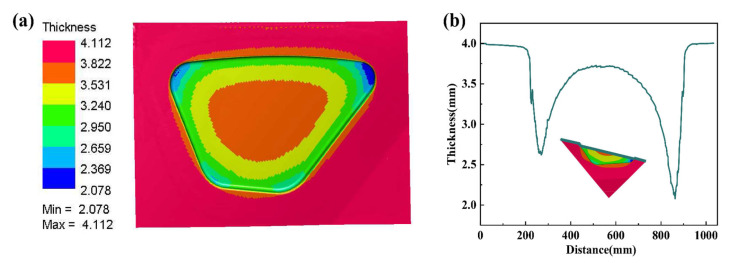
Simulation result (**a**) Numerical simulation cloud diagram of direct superplastic forming; (**b**) Thickness variation of the part along the section.

The rail train side window parts are directly subjected to superplastic forming, and the simulation results are shown in Figure 4. The blow-forming process of side window parts includes free bulging, blank to the surface of the die, and forming at the edge fillet. During the forming process, the central part of the blank adheres preferentially to the die. Due to viscous friction, the blank stays more in the non-part portion and no longer flows. This results in a smaller proportion of material filling the edge fillets, which increases the thinning at the fillets (Thickness is in mm).

### 3.2. Determination of Pre-Forming Die Parameters

**Figure 5:**
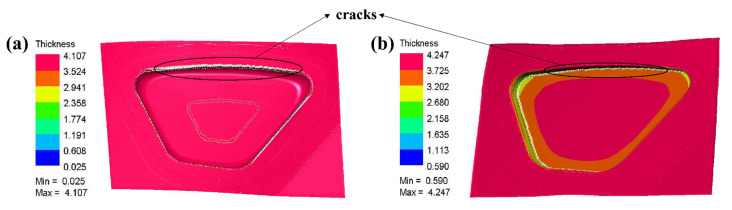
Effects of excessive punch depth and size; (**a**) Cracks in the top fillet due to excessive punch depth; (**b**) Cracks in the bottom fillet due to excessive punch size.

Superplasticity shows the comprehensive performance index of materials. Different materials have very different superplastic properties. There are many factors that affect the plasticity of materials. The general plasticity of materials is mainly affected by strain hardening. In the process of hot stamping, the depth H of the punch, the area ratio s of the punch, and the friction coefficient μ have a significant influence on the forming quality of the part. The process parameters should be selected in a suitable range. The effects of exceedingly large punch depth and area are shown in Figure 5. Excessive punch depth (30 mm) causes cracks in the top fillet, as shown in Figure 5a, and excessive punch area (80%) causes cracks in the bottom fillet, as shown in Figure 5b. Most of the blank is stuck to the bottom, which makes it difficult to obtain enough material when forming the edge fillet. The small space within the cavity for blow forming defeats the original purpose of preforming to overcome the effects of viscous friction.

Superplastic property is an important index to measure the high-temperature formability of materials. Due to the excellent application properties of aluminum alloys, their mechanical properties, especially high-temperature mechanical properties, have been widely studied. However, these studies are based on traditional deformation processes, such as the constant pressure method and the constant strain rate method. During the simulation, “H” defines as 20 mm, 17 mm, 14 mm, 11 mm, and 8 mm, respectively; “S” is defined as 64%, 52%, 40%, 28%, and 16%, respectively; μ is defined as 0.3, 0.25, 0.2, 0.15 and 0.1, respectively. Hot stamping was carried out under different conditions, and then the blow forming was carried out. Considering that thickness is influenced by a number of experimental factors, the Central Composite designs (CCD) model was used for the experimental design, and a Response Surface Methodology (RSM) was used to model the thickness as a function of the factors. CCD is the most commonly used response surface design test. The CCD is a factor or local factor consisting of a central point and extended by a series of pivot points. It can model response variables with curves by adding center and pivot points to a completed factorial design. An experimental design table with three factors and five levels was established in the CCD model. In the model, input variables *A*, *B*, and *C* represent “H,” “S,” and “μ,” respectively, and the r value is used as the response. The experimental design and results are shown in Table 2. The response value R represents the minimum thickness of the side window. The larger the response value, the better the forming quality of the part:

(12) $$s=\frac{s_{1}}{s_{2}}$$

where *s*_1_ represents the surface area of the pre-forming die and *s*_2_ represents the bottom surface area of the part.

The regression fitting analysis was performed on the test results, and the quadratic multiple regression equation of the minimum thickness was obtained based on the coding factor:

(13) $$\begin{array}{ccc} R & = & 1.94814+0.044583A+0.598264B+1.08667C\\ & + & 0.015278AB-0.015000AC+0.33333BC-0.001694A^{2}-1.12847B^{2}-6.30000C^{2} \end{array}$$

**Figure 6:**
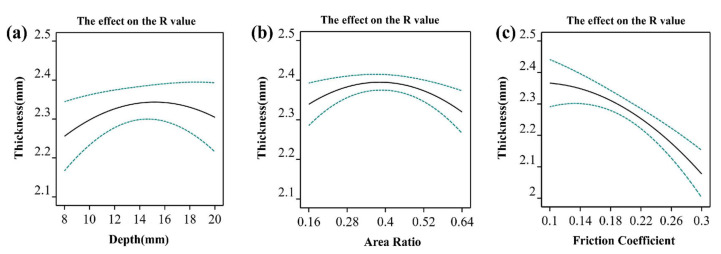
The effect of a single factor on the R-value. (**a**) Depth; (**b**) Area ratio; (**c**) Friction coefficient.

Both the predicted *R*^2^ = 0.9581 and the adjusted *R*^2^ = 0.9204 are reasonable; the difference is less than 0.2. Model F-value = 25.43 means the model is significant. The probability of such a large F value (F significance test for the CCD model) due to noise is only 0.01%, indicating a reasonable experimental design. The effects of single factors on the R-value were explored separately, as shown in Figure 6. The depth factor is shown in Figure 6a, and the area ratio factor is shown in Figure 6b, the friction coefficient factor is shown in Figure 6c.

**Figure 7:**
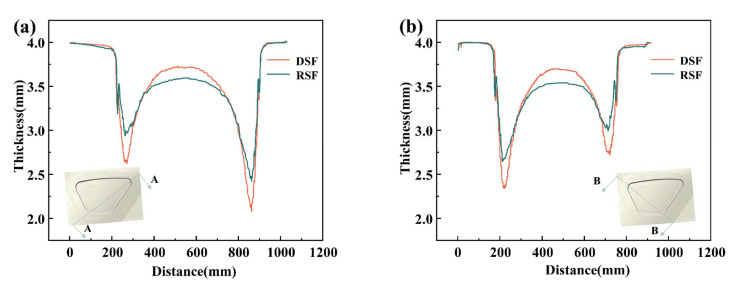
Thickness distribution on lines at the center of the fillets (**a**) Section A; (**b**) Section B.

Through the Numerical Optimization Algorithm, the best three parameters are determined: *A* = 14.411 mm, *B* = 0.378, and *C* = 0.1. By using this set of parameters, the minimum thickness of the formed part can be obtained as 2.424 mm. The thickness of the points on the connection line can be obtained by connecting the center points of the diagonal fillet of the formed part. The thickness distribution of the parts using both DSF and RSF forming methods is shown in Figure 7. The thicknesses of the four fillets of the formed parts corresponding to the RSF process are optimized. After the pre-forming process, the maximum thinning rate is reduced from 48.05% to 39.4%. The thickness of the finished part is also more evenly distributed on the whole.

The energy spectrum scanning results are shown in Table 3. In the basic phase, the average content of the Al element is 92.33%, the average content of the Mg element is 4.23%, and the average content of the Mn element is 0.91%. In the precipitated phase, the average content of Al is 91.72%. The average content of the Mg element is 3.89%, and the average content of the Mn element is 1.23%. This is almost consistent with the energy spectrum test results of the basic blank, which indicated that the phase types in the blank have not changed after blow forming.

Using finite element numerical simulations, the forming limits of box-shaped parts in different forming conditions are determined. The sidewall height H of the box was measured, and the measured results are shown in Table 4. When the temperature was 480 °C, and the pressure was 1.7 MPa, the maximum sidewall height was 26.7 mm. When the temperature was 510 °C, and the pressure was 1.7 MPa, the maximum sidewall height was 27.6 mm. During the superplastic stretching process, the crystals undergo dynamic recovery with the increase in temperature. The metal was softened to a certain extent, and the tensile load was reduced. At the same time, the increase in temperature increased the free energy of atoms, which enhanced the ability of grain boundary sliding and diffusion, thereby improving its properties.

### 3.3. Pressure Curve for Superplastic Forming

**Figure 8:**
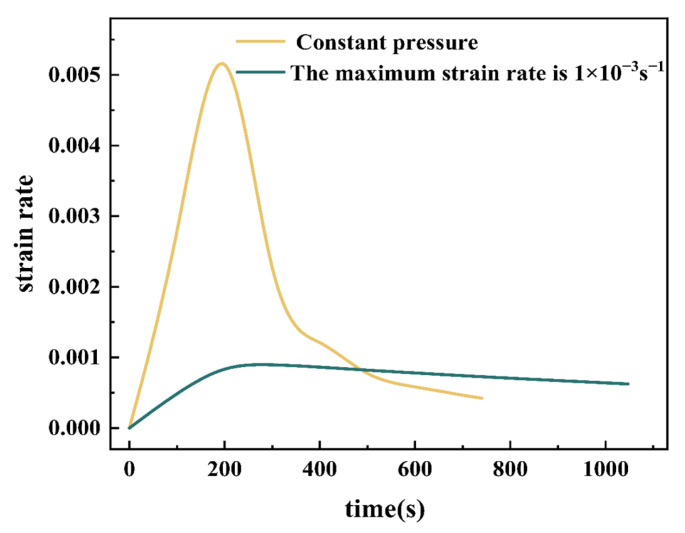
Maximum strain rate at constant pressure vs. maximum strain rate of 1 × 10^−3^ s^−1^.

Under the premise of the optimum pre-formed depth, area ratio, and friction coefficient, the forming quality of the die is improved. Compared with direct superplastic forming, the thickness of the sharp fillet and the thickness around the fillet is increased. At the same time, the underside area, which is not part of the use, decreases in thickness. However, as the thickness increase at the sharpest point of the fillet is extremely limited, so the pressure curve during superplastic forming is optimized to obtain better forming results. Gas pressure (N_2_) and strain rate during part forming are two important elements in determining the pressure curve. The magnitude of the strain rate affects the deformability of the blank. Excessive strain rates make dynamic recrystallization of grains difficult. This leads to coarse grain size and non-uniformity in the formed part. On the contrary, when the strain rate is too low, the tendency of recrystallized grains grows abnormally, resulting in reduced strength. Furthermore, as the increased heating time may cause abnormal grain growth, an appropriate strain rate should be selected. As mentioned above, the tensile properties are better at strain rates of 1 × 10^−3^ s^−1^ and 5 × 10^−4^ s^−1^. They can meet production quality requirements. In order to improve productivity, the strain rate during gas forming was controlled to below 1 × 10^−3^ s^−1^ in the forming study. Shown in Figure 8 is the curve of the maximum strain rate when the part is loaded with constant pressure and the maximum strain rate curve with a controlled maximum strain rate of 1 × 10^−3^ s^−1^. It can be seen that the maximum strain rate at a certain stage of the forming process is greater than 1 × 10^−3^ s^−1^ and less than 1 × 10^−2^ s^−1^ when constant pressure is used as the forming driving force. This situation indicates that there is a time period in the forming process when the superplastic forming performance is poor, and the final forming effect has a certain negative impact.

**Figure 9:**
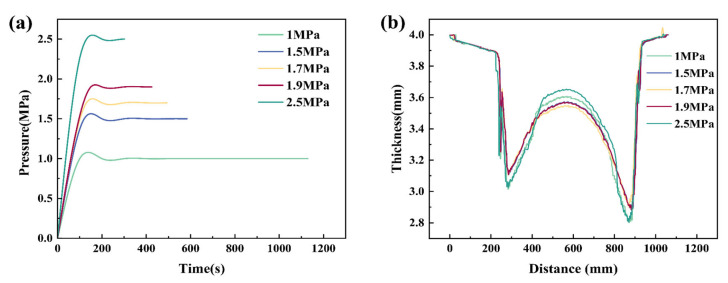
(**a**) Pressure curves, (**b**) Thickness distribution on the connection between the center of the sharp fillet.

The method of controlling the maximum strain rate during the forming process will result in longer forming times. An appropriate increase in the rated pressure will reduce the forming time of the part while maintaining production quality. The maximum pressure that can be achieved by the superplastic forming equipment used in the experiments is around 2.5 MPa, so the rated pressures are set as 1.0 MPa, 1.5 MPa, 1.7 MPa, 1.9 MPa, and 2.5 MPa, respectively. The pressure curves at different rated pressures are shown in Figure 9a. As shown in Figure 9b, the thickness distribution on the line at the center of the formed part fillet corresponds to the different pressure curves. With the increase in pressure, the time required to form the part is shorter, and the forming efficiency is higher. When the pressure is too low, the forming height is limited, and the fillet filling stage, where the deformation is difficult, takes a long time. This causes grain growth, resulting in reduced forming properties and higher thinning rates. When the pressure is too high, the balance between strain hardening and strain softening is difficult to maintain, although the forming efficiency is higher. The tendency for strain hardening is more pronounced during the forming process. Good forming results are not achieved. At a forming pressure of 1.7 MPa, the forming part has the lowest thinning rate and the most uniform thickness distribution. After forming, the thinning of the part is controlled to less than 30%, and the forming time is about 8 min, which can achieve the production efficiency requirements.

**Figure 10:**
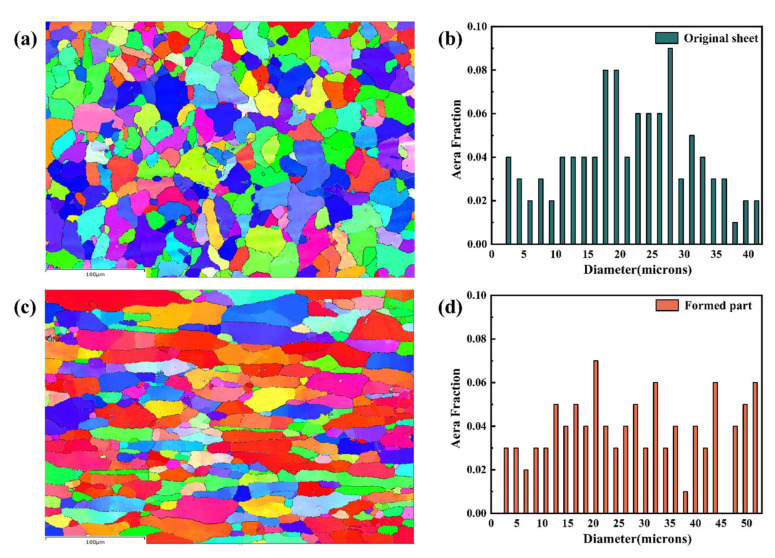
(**a**) EBSD diagram of the original 5083Al sheet; (**b**) Grain size statistics of the original 5083Al sheet, (**c**) EBSD diagram of 5083Al after blow forming; (**d**) Grain size statistics after blow forming.

To study the grain size evolution before and after deformation, EBSD was performed at 20 kV, a working distance of 22 mm, and an inclination angle of 70°, with a scan step of 1 μm. The mapping data were measured in the RD-TD plane, and all images had a resolution rate above 90%. The grain size statistics of 5083Al before and after blow forming are shown in Figure 10. After blow forming, the grain size of 5083Al increased to a certain extent. By calculation, the average grain size of 5083Al after superplastic forming was 28.92 μm, and the average grain size when undeformed was 21.55 μm. The higher the temperature, the greater the atomic diffusion coefficient, and the easier it is for grain boundaries to migrate and for grains to coarsen. During deformation, strain can lead to dissimilar dislocation pairing and subcrystalline aggregation, which increases the average size of the grain.

## 4. Conclusions

Recently, the superplastic forming process of aluminum alloys has received extensive attention. The rapid superplastic forming process has the advantage of a shorter forming time and more uniform thickness distribution compared to the direct superplastic forming process. Therefore, this paper investigated the rapid superplastic forming process for the rail vehicle side window made of 5083Al alloy. Compared with previous studies, the data of this study is relatively complete and innovative. The following conclusions were reached:

1. High-temperature tensile tests were carried out on 5083Al alloy, and the best deformation conditions for this material were obtained at a temperature of 510 °C and a strain rate of 5 × 10^−4^ s^−1^ when the elongation reached 221.7%;
2. The influences of parameters of the pre-forming die on the thinning rate were investigated using the Response Surface Methodology (RSM). The optimum parameters of the pre-forming die were a depth of 14.411 mm, an area ratio of 0.378, and a friction coefficient of 0.1. The optimal parameters are detected by finite element simulation;
3. Based on the optimization results of the pre-forming die, the simulations of the rapid superplastic forming were performed at different rated pressures. With a controlled maximum strain rate of 1 × 10^−3^ s^−1^, the best forming results were obtained when the rated pressure was 1.7 MPa, yielding a thinning rate of less than 30%;
4. Analysis of the microstructure of the blank before and after blow forming showed that there was no change in the phase-type and the average grain size increased from 21.55 μm to 28.92 μm.

## Figures and Tables

**Table 1:** Chemical composition of 5083Al.

Element	Content (wt%)
Si	≤0.40
Mg	4.0–4.9
Zn	≤0.25
Mn	0.4–1.0
Ti	≤0.15
Cr	0.05–0.25
Fe	0–0.4
Al	Bal.

**Table 2:** Experimental design and results.

A (mm)	B (%)	C	R (mm)
14	16	0.2	2.278
11	28	0.15	2.379
14	40	0.2	2.282
17	52	0.15	2.378
11	52	0.25	2.202
14	40	0.2	2.346
14	64	0.2	2.266
14	40	0.2	2.346
14	40	0.3	2.137
8	40	0.2	2.277
14	40	0.2	2.346
11	52	0.15	2.347
17	28	0.25	2.203
20	40	0.2	2.275
14	40	0.2	2.346
17	52	0.25	2.201
11	28	0.25	2.203
14	40	0.1	2.411
17	28	0.15	2.365
14	40	0.2	2.346

**Table 3:** Energy spectral scan results.

Element	Basic Phase	Precipitated Phase
Al (%)	92.33	91.72
Mg (%)	4.23	3.89
Mn (%)	0.91	1.23
Other (%)	2.53	3.16

**Table 4:** Box-shaped part sidewall height measurement.

Temperature T (°C)	Pressure P (MPa)	Sidewall Height H (mm)
480	1.5	24.6
480	1.7	26.7
480	1.9	25.1
510	1.5	24.9
510	1.7	27.6
510	1.9	22.3

## Data Availability

Datasets generated and/or analyzed during the current study are available from the corresponding author on request.
